# Immature platelet fraction and thrombopoietin in patients with liver cirrhosis: A cohort study

**DOI:** 10.1371/journal.pone.0192271

**Published:** 2018-02-13

**Authors:** Philip Rauber, Frank Lammert, Katharina Grotemeyer, Beate Appenrodt

**Affiliations:** 1 Department of Neurology, Saarland University Medical Center, Homburg/Saar, Germany; 2 Department of Medicine II, Saarland University Medical Center, Homburg/Saar, Germany; Chang Gung Memorial Hospital Kaohsiung Branch, TAIWAN

## Abstract

**Background and aims:**

Thrombocytopenia occurs frequently in patients with cirrhosis. The immature platelet fraction (IPF%) is measured to differentiate the causes of thrombocytopenia. To date the relevance of thrombopoietin (TPO) in the context of cirrhosis is unknown. The aim of our study was to investigate the cause of thrombocytopenia in patients with liver cirrhosis by measuring IPF%, TPO and spleen size. In addition we examined the use of IPF% to evaluate the severity of cirrhosis and its complications.

**Methods:**

Overall, we included 88 in-patients with cirrhosis in our study. The collected data comprises current health status, blood parameters, severity of cirrhosis evaluated by Child-Pugh score and MELD score, spleen diameter, ascites and esophageal varices. The IPF% was measured using an automatic hematology analyzer. TPO was measured with ELISA.

**Results:**

IPF% (p = 0.003) and spleen diameter (p = 0.001) were significantly higher in patients with thrombocytopenia. There was no significant difference in TPO between patients with and without thrombocytopenia. The mean values of IPF% varied significantly (p = 0.044) in Child-Pugh stages. IPF% was significantly (p = 0.005) elevated in patients with esophageal varices. Moreover, IPF% higher than 3.85% displayed sensitivity of 76.6% and specificity of 52.4% with an area under receiver operating curve characteristics of 0.669 for the presence of esophageal varices.

**Conclusion:**

On closer examination of the three compartments known to have an influence on platelet count splenomegaly seems to be the major cause of thrombocytopenia in patients with cirrhosis according to current knowledge. Higher IPF% in patients with thrombocytopenia indicates peripheral consumption of platelets. The relation between spleen diameter and platelet count indicates the spleen to be the major place of platelets’ consumption. TPO did not differ between patients with and without thrombocytopenia. Furthermore, we cannot exclude an influence of impaired thrombopoietin synthesis on platelet counts. The association between IPF% and platelet count suggests that there is physiological regulation of platelets in patients with cirrhosis. In our study IPF% is associated with esophageal varices and the stage of cirrhosis. Further studies are needed to confirm these results.

## Introduction

Thrombocytopenia is a common complication in patients with liver cirrhosis and its pathogenesis is multifactorial. [1,2] Previous studies have reported the splenic sequestration of platelets to be a consequence of an enlarged spleen due to portal hypertension. [3] Furthermore recent studies propose that decreased TPO production in the liver and an impaired platelet production in the bone marrow are additional factors. [4,5] Increasing TPO levels and platelet recovery after orthotopic liver transplantation confirm these results as well as the availability of the TPO receptor agonist eltrombopag, which increases platelet counts in patients with liver cirrhosis caused by chronic hepatitis c virus infection. [6–9] In contrast, other studies have not been able to verify the associations between liver cirrhosis and decreased TPO production. [10–12] There are further factors that have an influence on platelet counts, such as anti platelet autoantibodies leading to shortened lifespan by removal via the reticuloendothelial system or chronic alcoholism, leading to the suppression of platelet production in the bone marrow. [13,14] Consequently the exact pathogenesis of thrombocytopenia in patients with liver cirrhosis is still unclear.

The interpretation of serum TPO levels is complex. [4,12,15,16] The term reticulated platelets describes immature platelets that contain remnants of RNA. [17] In analogy to reticulocytes that are a valid marker of erythropoesis in bone marrow, reticulated platelets, measured as immature platelet fraction using an automated blood cell analyzer, reflect thrombopoesis. [18] The immature platelet fraction is low when there is impaired bone marrow function and is high in case of peripheral consumption or destruction of platelets. [19] Therefore, it can be used to differentiate between the etiologies of thrombocytopenia. [20] Reticulated platelets have been reported to be lower in patients with liver cirrhosis and thrombocytopenia as compared to patients without. [21] Furthermore, the stage of chronic liver disease can be assessed by measuring the immature platelet fraction. [22] In addition, the development of complications in patients with cirrhosis such as variceal bleeding, ascites, spontaneous bacterial peritonitis or hepatorenal syndrome is associated with poor prognosis. Hence the importance of non-invasive markers for such complications has been the focus of many research studies for many years. [23–27] Low platelet counts are associated with esophageal varices, community-acquired spontaneous bacterial peritonitis and mortality. [28–31] There are however no studies investigating the role of the immature platelet fraction. Hence, the aim of our study was to investigate the cause of thrombocytopenia by measuring immature platelet fraction, thrombopoietin and spleen size. In addition, we assessed whether immature platelet fraction could be used to evaluate the severity of cirrhosis and its complications.

## Patients and methods

All data were obtained from participants that provided written informed consent. The following data were obtained: current health status, severity of cirrhosis evaluated by Child-Pugh score and Model for End-stage Liver Disease (MELD) score, spleen diameter, ascites and esophageal varices. The study was approved by the ethics committee of the Ärztekammer des Saarlandes (Ref. 271/11). In total, 88 in-patients with liver cirrhosis took part prospectively between March 2013 and February 2014 (30 females, 58 males; mean age 61±12 years). Exclusion criteria included: absence of liver cirrhosis, age < 18 years, acute bleeding, bone marrow disease or malignant disease (with the exception of hepatocellular carcinoma) with radio- or chemotherapy. Thrombocytopenia was defined as platelet count lower than 150.000/μl; subgroups were stratified into severe (lower than 50.000/μl), moderate (50.000–100.000/μl) and mild (100.000–150.000/μl). Etiology of cirrhosis, age and sex were recorded. Stage of liver cirrhosis, spleen diameter and ascites were assessed based on full history, clinical examination, abdominal ultrasound or magnetic resonance imaging. Esophageal varices were assessed by upper gastrointestinal endoscopy. Blood samples were collected from all patients for the following assays: complete blood count with platelet count and immature platelet fraction, thrombopoetin, albumin, bilirubin, international normalized ratio (INR), creatinin, cystatin C, c-reactive protein (crp), procalcitonin and n-terminal pro brain natriuretic peptide (nt-proBNP). Child-Pugh and MELD scores were calculated. The blood count assays were performed on a Sysmex XE-5000 hematology analyzer (SYSMEX CORPORATION, Kobe, Japan). The immature platelet fraction reference range is 1.1–6.1%. [19] Thrombopoietin measurement was performed with Quantikine Human TPO immunoassay (R&D Systems, Minneapolis, USA). The thrombopoietin reference range is 70-100ng/l. [32] Ascites was assessed by abdominal ultrasound and stratified into mild, moderate and large. Hepatorenal syndrome type I and II were defined according to criteria by the German S3 guideline by Gerbes et al. [33]. Esophageal varices were defined according to criteria by Paquet [34].

Statistical analyses were performed using SPSS® software (IBM SPSS Statistics 20). Correlations between continuous variables were assessed using Spearman´s rank correlation coefficient. Differences between groups were compared using independent Student´s t-test for continuous variables and one-way analysis of variance (ANOVA). Receiver operating characteristic (ROC) curves were plotted to evaluate the diagnostic significance of potential predictive parameters for complications in patients with liver cirrhosis. Alpha level for statistical significance was set to 0.05 for all analyses. Data are presented as mean ± SD.

### Results

Table 1 shows the characteristics of the patients in the study.

**Table 1:** Patient characteristics.

Age (years) ± SD	61 ± 12
Women/men (%)	30 / 58 (34.1 / 65.9)
Child-Pugh A / B / C (%)	44 / 35 / 9 (50 / 39.8 / 10.2)
Spleen diameter (mm) ± SD	135 ± 28
Platelet count (x 10^9^ / l) ± SD	132 ± 80
IPF (%) ± SD	5.8 ± 3.6
Thrombopoietin (ng / l) ± SD	67 ± 69
Ascites (%)	50 (56.8)
- Mild / moderate / large	20 / 3 / 27 (22.7 / 3.4 / 30.7)
Spontaneous bacterial peritonitis (%)	7 (8)
Hepatorenal syndrome (%)	9 (10.2)
- HRS type I / HRS type II	3 / 6 (3.4 / 6.8)
Esopageal varices (%)	48 (54.5)
- Stage I / stage II / stage III	33 / 12 / 3 (37.5 / 13.6 / 3.4)

**Fig 1:**
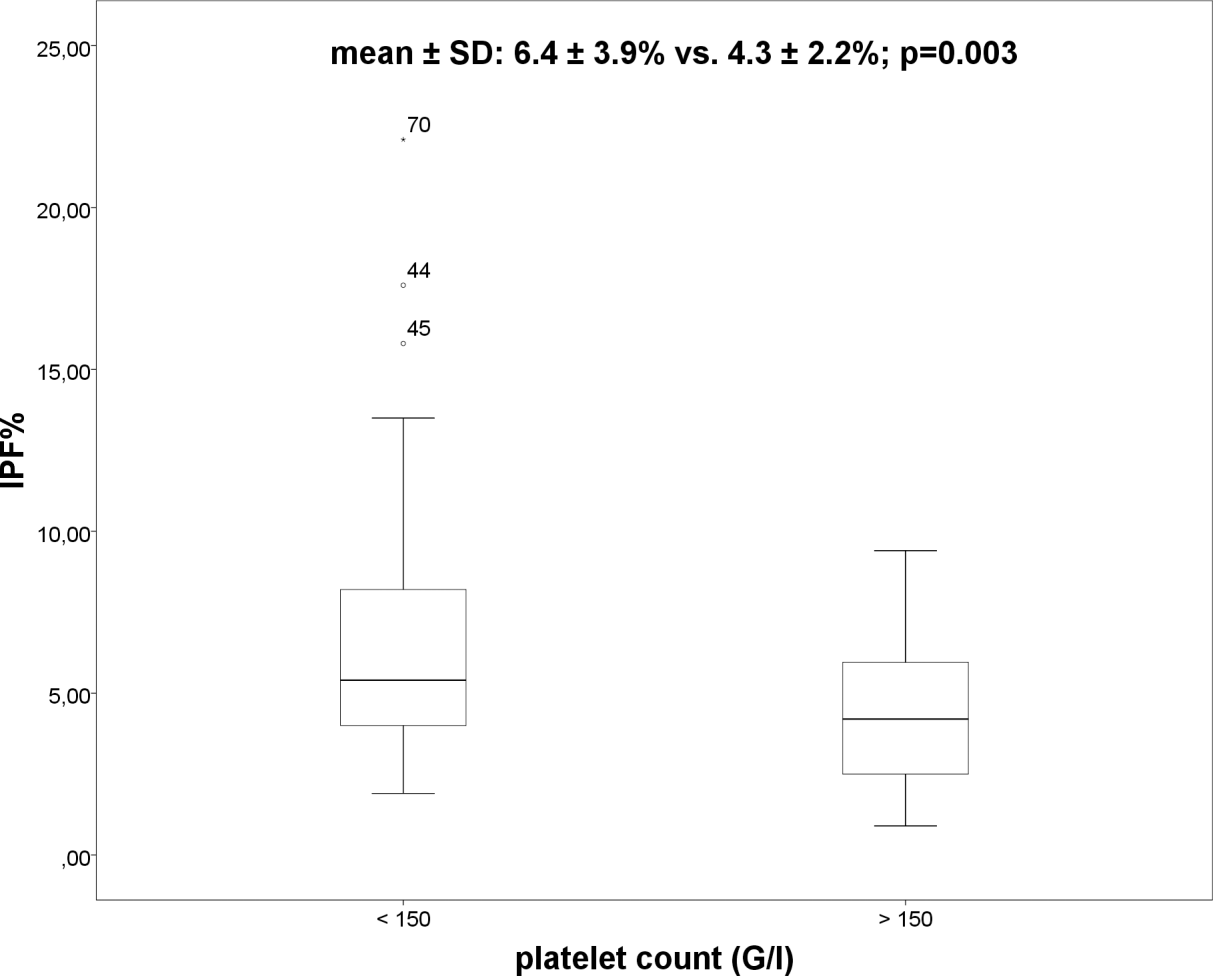
Boxplot: IPF% and platelet count.

**Fig 2:**
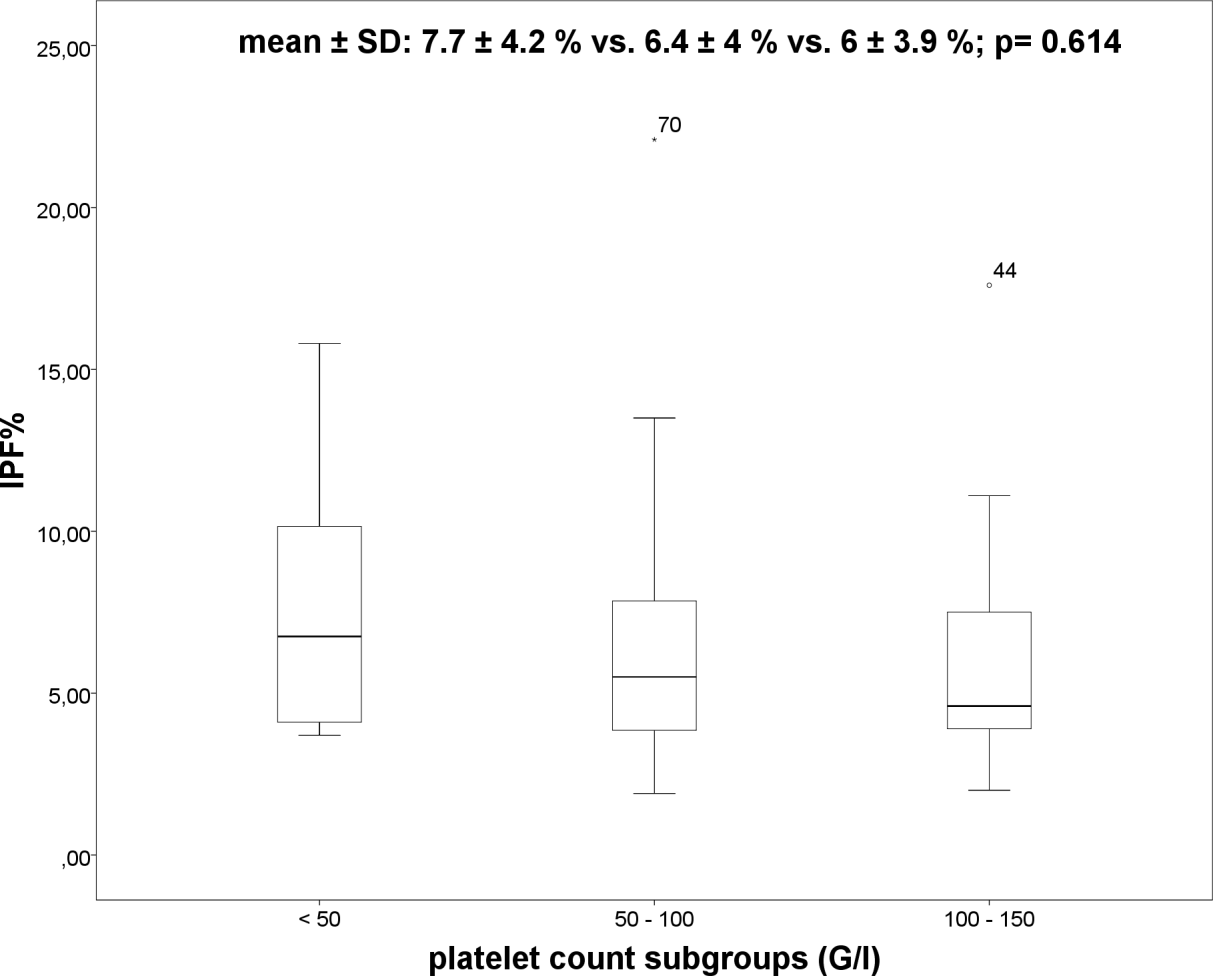
Boxplot: IPF% and the platelet count subgroups.

**Fig 3:**
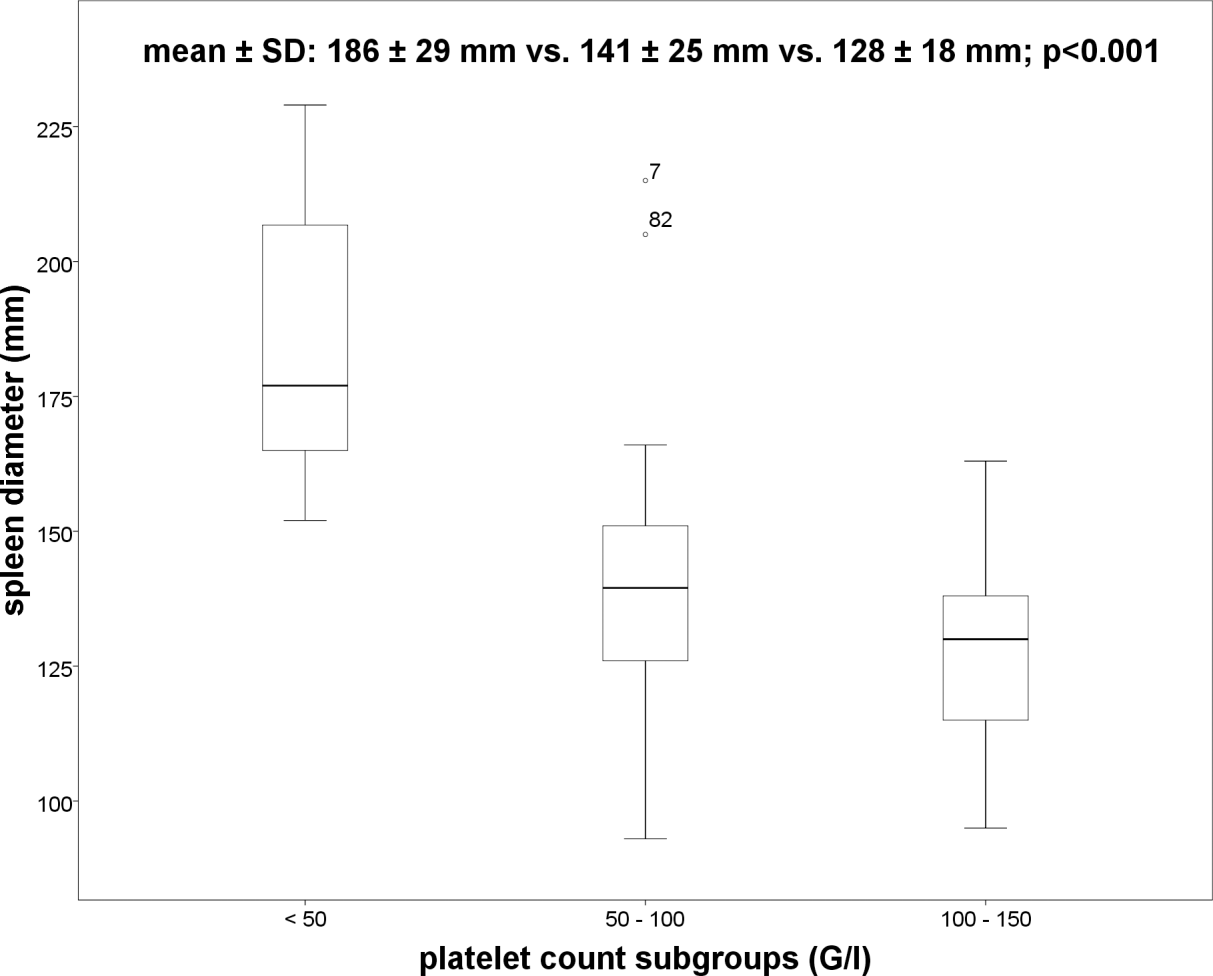
Boxplot: Spleen diameter and platelet count subgroups.

Overall, 59 patients had thrombocytopenia and 73 patients presented with splenomegaly. In patients with thrombocytopenia IPF% (6.4 ± 3.9% vs. 4.3 ± 2.2%; p = 0.003) (Fig 1) and spleen diameter (142 ± 29mm vs. 120 ± 20mm; p = 0.001) were significant higher as compared to patients without thrombocytopenia. No significant differences in IPF% between the subgroups of thrombocytopenia (severe, moderate and mild) using ANOVA were observed (7.7 ± 4.2% vs. 6.4 ± 4% vs. 6 ± 3.9%; p = 0.614) (Fig 2). However, significant differences were demonstrated in spleen diameter between the three subgroups of thrombocytopenia (Fig 3) using ANOVA (186 ± 29mm vs. 141 ± 25mm vs. 128 ± 18mm; p<0.001). Bonferroni correction confirmed significant differences in spleen diameter between severe and moderate thrombocytopenia (p = 0.000) and severe and mild thrombocytopenia (p = 0.000) but not between moderate and mild thrombocytopenia (p = 0.171).

**Fig 4:**
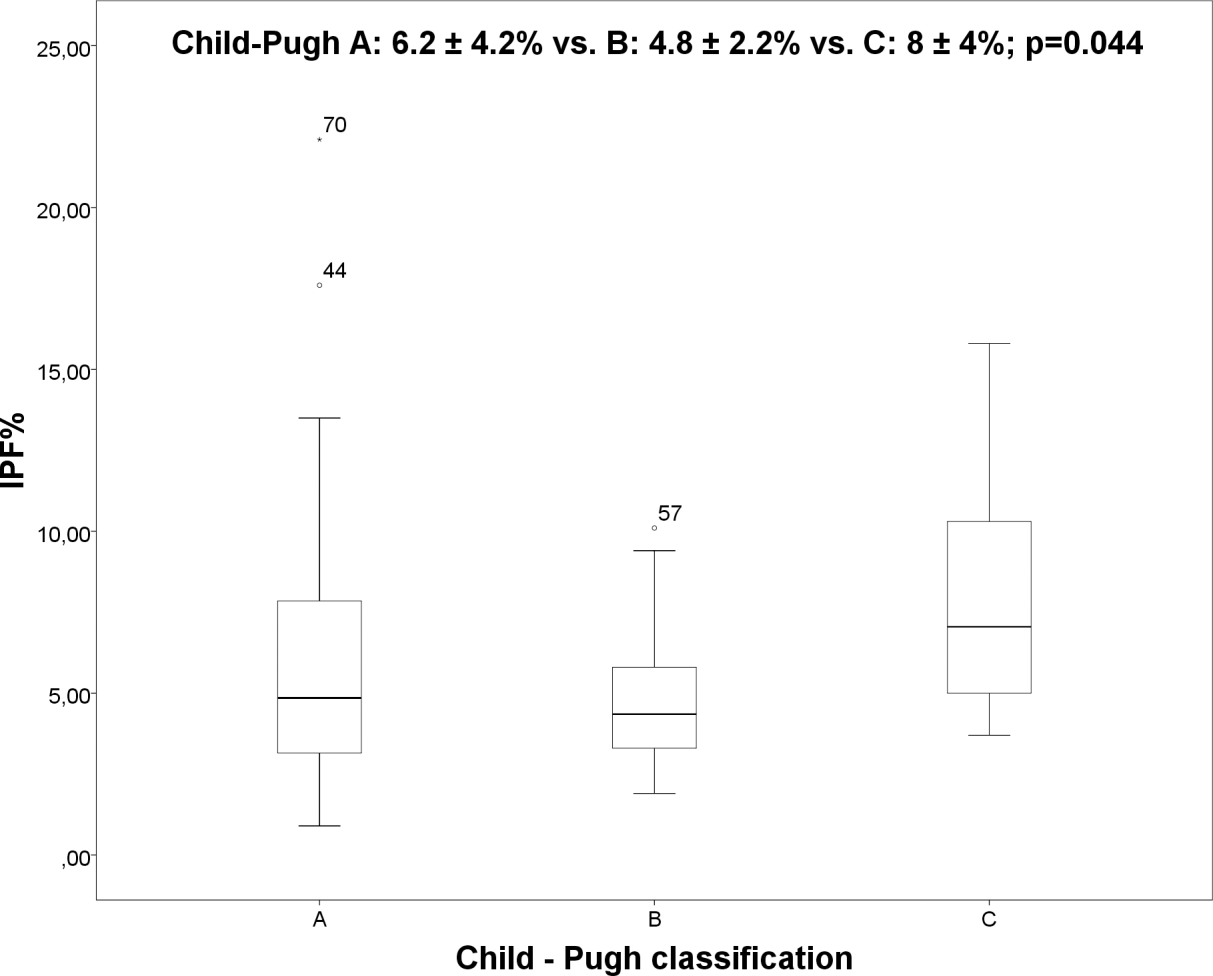
Boxplot: IPF% and Child-Pugh classification.

There were no significant differences in TPO concentrations (70 ± 65ng/l vs. 62 ± 76ng/l; p = 0.638) between patients with and without thrombocytopenia. Inverse correlations were observed between platelet count and IPF% (r = -0.341; p = 0.001) as well as between platelet count and TPO (r = -0.235; p = 0.035), and between platelet count and spleen diameter (r = -0.535; p<0.001). Neither IPF% and TPO (r = 0.035; p = 0.755) nor IPF% and spleen diameter (r = 0.023; p = 0.419) were correlated. There were no differences for IPF%, TPO, platelet count and spleen diameter in the two groups of patients with chronic hepatitis c virus infection and alcohol abuse as etiology for cirrhosis. In our study, the IPF% was associated with stage of cirrhosis and esophageal varices. Mean IPF% varied significantly in patients with different Child-Pugh stages using ANOVA (Fig 4) (Child-Pugh A: 6.2 ± 4.2% vs. B: 4.8 ± 2.2% vs. C: 8 ± 4%; p = 0.044). Bonferroni correction showed no significant differences between the subgroups (Child A and B: p = 0.272; Child A and C: p = 0.510 and Child B and C: p = 0.064).

**Fig 5:**
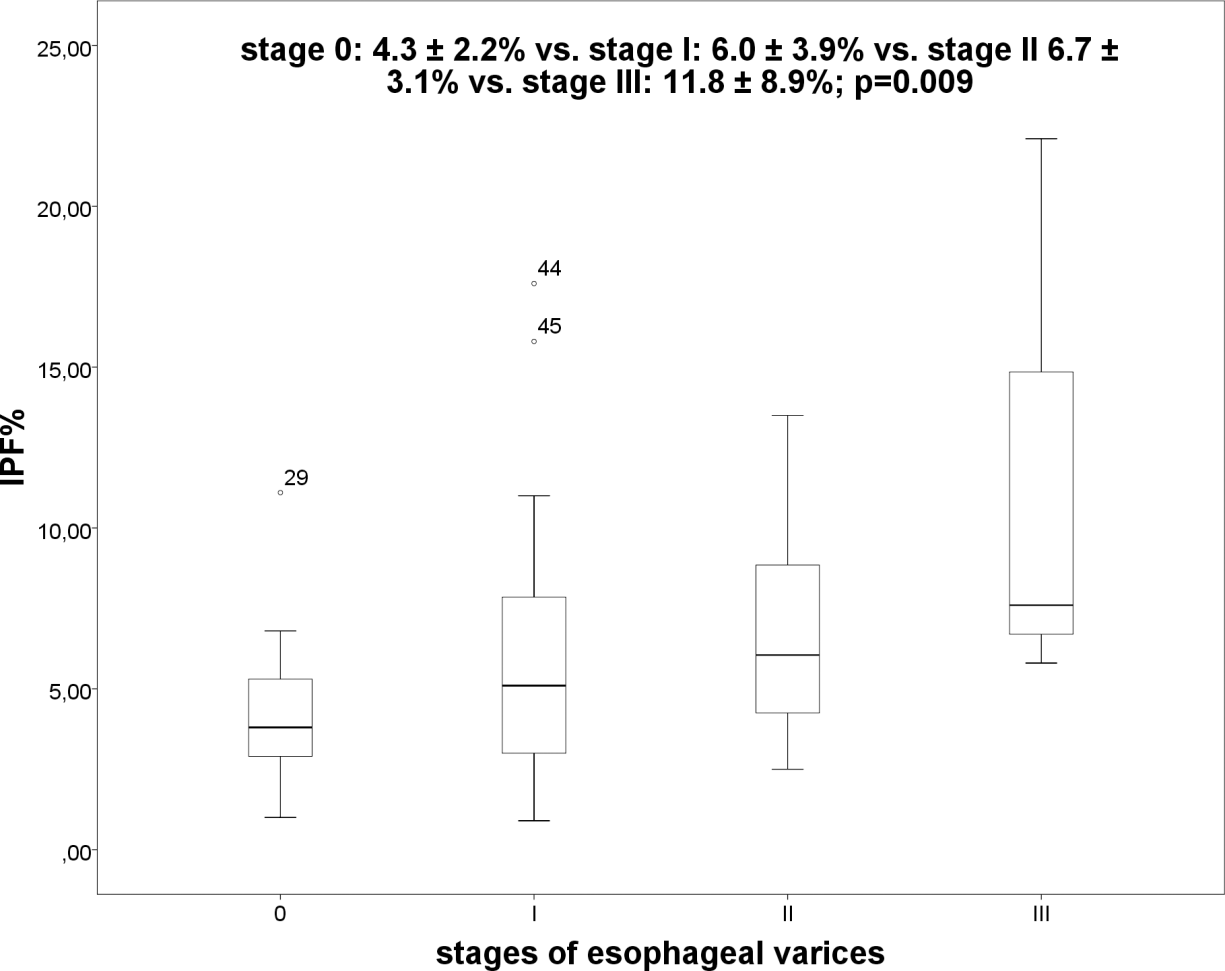
Boxplot: IPF% and stage of esophageal varices.

We compared IPF% in patients with MELD score ≤ or >15, however, the differences were not significant (p = 0.559). Furthermore, the IPF% was significantly (p = 0.005) elevated in patients with esophageal varices (without EV: 4.3 ± 2.2% vs. EV: 6.6 ± 4.2%), and there was a significant (p = 0.009) difference between the stages of esophageal varices (Fig 5) (stage 0: 4.3 ± 2.2% vs. stage I: 6.0 ± 3.9% vs. stage II 6.7 ± 3.1% vs. stage III: 11.8 ± 8.9%). Bonferroni correction illustrated significant differences between stage 0 and stage III (p = 0.008), however, there was no significant difference between the other stages (stage 0 and I, p = 0.569; stage 0 and II, p = 0.470, stage I and II, p = 1.000; stage I and III, p = 0.059; stage II and III, p = 0.178).

**Fig 6:**
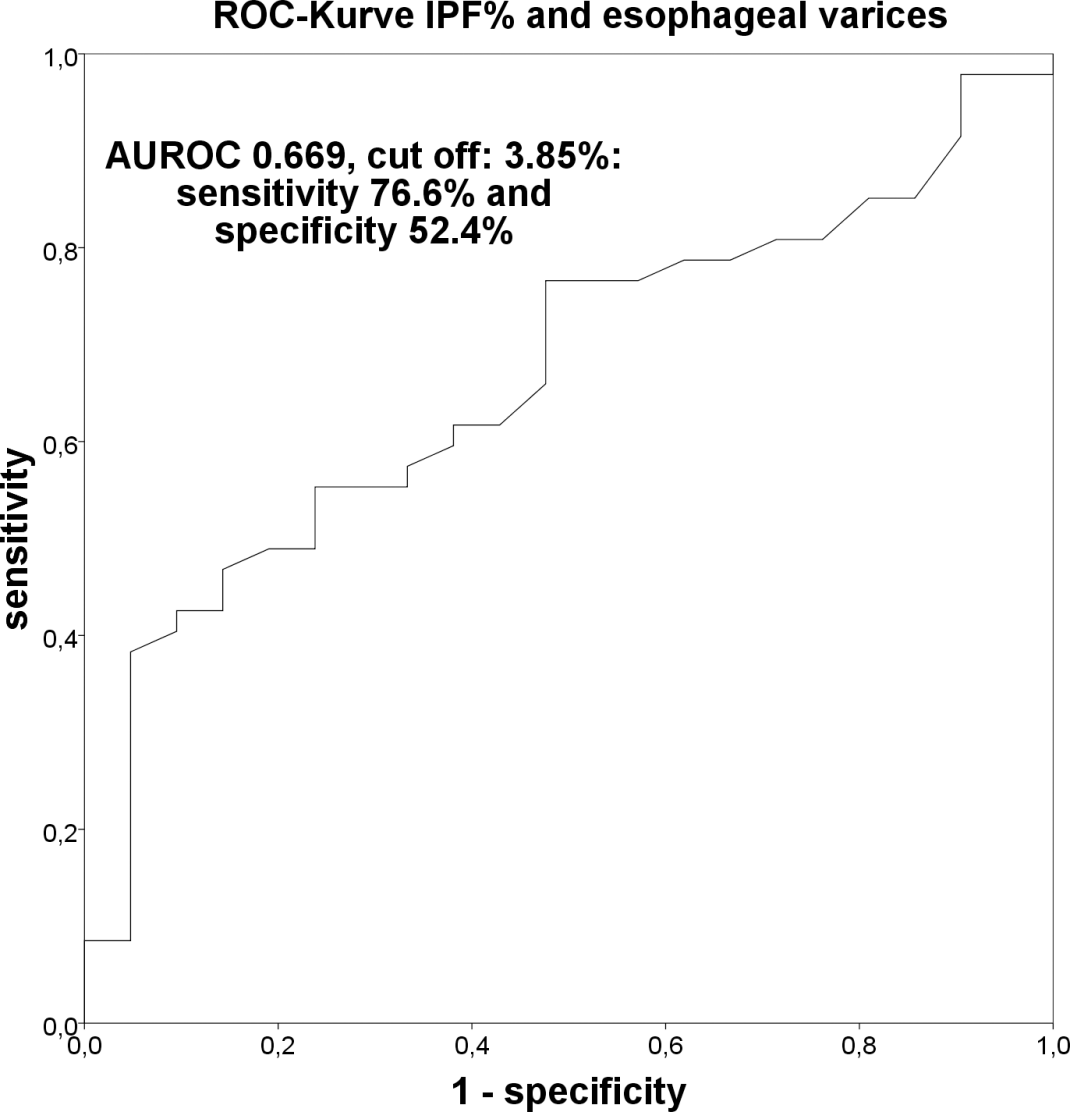
Receiver operating characteristics curve: IPF% and esophageal varices.

IPF% higher than 3.85% had a sensitivity of 76.6% and a specificity of 52.4% with an area under the ROC curve of 0.669 for the presence of esophageal varices (Fig 6).

There were no significant differences for IPF% in the following comparisons: patients with and without c-reactive protein (crp) higher than 0.5 mg/dl (p = 0.769), in patients with and without leucocyte counts higher than 10 x 10^9^/l (p = 0.546), in patients with and without procalcitonin (pct) higher than 0.5 ng/ml (p = 0.795) and in patients with and without spontaneous bacterial peritonitis (p = 0.470).

## Discussion

Thrombocytopenia is a common hematological abnormality that is either caused by increased sequestration and destruction of platelets or by decreased production. In patients with liver cirrhosis the pathogenesis is multifactorial but remains to be fully elucidated. Currently decreased platelet production in bone marrow and inadequate thrombopoietin production are purported to be the major causes. [5,35] In this context the immature platelet fraction (IPF%) might be a useful parameter for differential diagnosis. [20] The high IPF% in patients with thrombocytopenia in our study indicates peripheral consumption of platelets without bone marrow failure. [19] Therefore, on closer examination on whether the three compartments have an influence on platelet count, the significant relation between spleen diameter and platelet count indicates the spleen to be the place of consumption and destruction of platelets in our study, without taking into consideration the possible influence of the recently discovered Ashwell-Morell receptor. [36] These results are consistent with the studies by Zucker et al. [11] and Tana et al. [37]. The inverse correlation between IPF% and platelet count in our study points to the physiological regulation of platelets in patients with cirrhosis. [38] These results are in contrast to a recent study that observed no correlation between platelet count and IPF% and proposed that this could indicate development of cirrhosis in patients with chronic liver disease. [22] Prior to the current study, the association between IPF% and stage of cirrhosis had not been investigated. One limitation of our study is the low number of patients with Child-Pugh stage C, and thus the physiological regulations might not function as they should with progressing cirrhosis. Indeed, serum TPO concentrations and platelet counts have been shown to increase post liver transplantation. [7] In patients with chronic viral hepatitis TPO levels are elevated and with progression from fibrosis to cirrhosis TPO synthesis in the liver decreases. [4] We observed no TPO differences in patients with and without thrombocytopenia, however, we cannot fully exclude an influence of impaired TPO synthesis on platelet counts. TPO serum levels are regulated by complex mechanisms. The TPO receptor c-MPL on platelets binds thrombopoietin [39] and the Ashwell-Morell receptor in the liver can affect platelet counts and TPO production via the JAK-STAT signaling pathway. [36] Our results are in line with those from Zucker et al. [11] and Temel et al. [10]. They reported no difference in TPO serum levels between patients with and without thrombocytopenia and no difference according to Child-Pugh stages. TPO levels in controls without cirrhosis however, were higher and platelet counts correlate physiologically with TPO. [10,11] Furthermore IPF% can be elevated in some other entities other than cirrhosis. In addition to the falsely elevated measurement of IPF% values in case of macrothrombocytopenia [40], IPF% is thought to be elevated in acute coronary syndromes [41] and acute bacterial infections [42]. In our study, patients with spontaneous bacterial peritonitis and all patients with elevated inflammatory parameters (elevated leucocyte count, c-reactive protein and procalcitonin) no significant difference in IPF% was observed when compared to patients without such conditions. There were no patients with acute coronary syndrome in our study.

The second aim of our study was to investigate the association between IPF% and stage of liver cirrhosis. Nomura et al. [22] found significantly elevated IPF% in patients with liver cirrhosis compared with healthy controls but did not differentiate between the stages of cirrhosis. The severity of cirrhosis in our study was measured by Child-Pugh classification and MELD score. Differences in MELD score were not significant. In addition to Child-Pugh stages IPF% was associated with the presence of esophageal varices. In the present study these associations are described for the first time and further studies are needed to confirm our results. Our results are in accordance with the studies by Temel et al. [10] and Schöffski et al. [12].

## Conclusion

In conclusion, the present study demonstrates the complex associations between liver cirrhosis and its complications. In our study splenomegaly appears to play the major role in the development of thrombocytopenia in patients with cirrhosis. The regulation of thrombopoesis also appears to be physiological. Closer examination of several compartments did not reveal a single cause of thrombocytopenia. Further studies are needed to investigate the changes of the Ashwell-Morell receptor function and the formation of desialylated platelets in patients with liver cirrhosis. Furthermore, the correlation between the TPO serum levels and IPF% in patients with liver cirrhosis has not been described before. In further studies longitudinal measurements of IPF% would be useful for monitoring progression and development of esophageal varices.

## Supporting information

S1 Table This table shows all data acquired from the patients in our study. (XLSX)
